# Impact of epidemic prevention policies on public vaccination willingness: empirical research in China

**DOI:** 10.3389/fpubh.2024.1329228

**Published:** 2024-07-03

**Authors:** Jie Zhong, Yue Zhuang, Miao Zhang

**Affiliations:** School of Safety Science and Emergency Management, Wuhan University of Technology, Wuhan, China

**Keywords:** COVID-19, prevention policy, vaccination willingness, risk perception, government

## Abstract

**Background:**

The sudden emergence of the COVID-19 pandemic in early 2020 posed an enormous threat to public health. Vaccination is currently recognized as the most cost-effective preventive and control measure against the COVID-19 pandemic worldwide and is the key to constructing a line of defense against the virus, while the epidemic prevention policies adopted by governments have an important impact on the protective behavior of the public. The purpose of this study is to analyze the mechanism by which the stringency of epidemic prevention policies affects public vaccination willingness and to explore the mediating effect of public risk perception.

**Methods:**

A cross-sectional survey was conducted among 387 adults from December 2022 to June 2023 in China. A multiple linear regression model was used to explore the impact of epidemic prevention policy stringency on public vaccination willingness, and a hierarchical regression model was used to test the mediating effect of public risk perception.

**Results:**

Our results showed that public vaccination willingness increased by approximately 45.5% for every one-unit increase in the stringency of the epidemic prevention policies, which shows that the stringency of epidemic prevention policies has a significant positive influence on public vaccination willingness. In addition, public risk perception increased by approximately 38.9% during the period of stringent government policies on epidemic prevention. For every one-unit increase in risk perception, public vaccination willingness increased by approximately 40.9%, and the relationship between the stringency of epidemic prevention policies and public vaccination willingness was partially mediated by risk perception.

**Conclusion:**

The stricter the epidemic prevention policies, the stronger the public vaccination willingness; risk perception plays a mediating effect between the stringency of epidemic prevention policies and public vaccination willingness. This finding is particularly important for exploring and analyzing the factors influencing public vaccination willingness and for improving public health.

## 1 Introduction

According to the World Health Organization (WHO), vaccination prevents approximately 2 to 3 million deaths each year (1). In the 21st century, preventive strategies centered on vaccination have the potential to help effectively control and eradicate a number of infectious diseases that pose a serious threat to human life and which can debilitate individuals who contract these diseases. The WHO declared COVID-19 to be a pandemic on March 11, 2020 (2); it was a significant public health emergency with a high transmission rate and wide infection range. According to the WHO, as of December 31, 2023, there had been almost 99.3 million confirmed cases of COVID-19 in China, with 121,900 deaths (3).

In the context of the global COVID-19 pandemic, promoting mass COVID-19 vaccination and increasing public vaccination rates are the top priorities for global novel coronavirus prevention and control (4). According to the Joint Committee on Vaccination and Immunization (JCVI), individuals who are 65 years of age or older, high-risk groups, and healthcare workers are the priority populations for vaccination (5). For individuals and families, vaccination can prevent the disease from occurring, achieve the effect of active immunization, and reduce the chances of transmission of COVID-19; for the collective population and society, increasing the vaccination rate can establish a herd immunity barrier, effectively blocking the spread of the disease.

However, vaccine adherence has always been one of the primary obstacles confronting the field of public health. Workplace vaccination campaigns have the potential to be a very useful public health tool to ensure vaccination adherence (6), but were underused during the COVID-19 pandemic. The COVID-19 pandemic has forestalled the painstaking but incremental progress made in the last decade to improve vaccine uptake (7). In the United States, after the national emergency declaration, the aggregate count for pediatric vaccine doses procured by Vaccine-for-Children (VFC) providers substantially declined (8). Similarly, the WHO recorded a 28-year reduction in global coverage for the Tdap vaccine (9). In Western countries, a number of anti-vaccine groups have been strongly resistant to vaccination, and as a result many countries have adopted mandatory vaccination policies. In the United States and Italy, for example, parents who do not comply with childhood vaccination schedules are barred from enrolling their children in public schools and daycare centers, and in some cases are required to pay penalties (10). Smoking, non-daily physical exercise, irregular medication adherence, and comorbidities were found to be risk factors for COVID-19 vaccination among Chinese adults (11). On July 20, 2023, the coverage rate of the first dose of the COVID-19 vaccine was 92.9% and the full vaccination rate was 90.5% among the whole population in mainland China (12). Although China currently leads the world in vaccination rates, it is still some way from achieving the goal of universal vaccination. Therefore, focusing on the phenomenon of COVID-19 vaccine unwillingness among the public and further promoting public vaccination are essential steps to enhance the immunization base of the whole society.

The global COVID-19 pandemic has elicited diverse reactions from governments worldwide. Even in recent times, when most governments have reopened and loosened most restrictions, the impact of government prevention policies has been the subject of much debate (13). Chinese government epidemic prevention policies are also intricately interconnected with public vaccination willingness. The government’s epidemic prevention policies have evolved through several stages, adapting to the changing circumstances in epidemic prevention and control and the government’s deepening understanding of the COVID-19 pandemic. At different times, the government’s epidemic prevention policies have varied in stringency. The public’s perceived level of risk also fluctuates, meaning that their epidemic prevention and control behaviors will also change accordingly (14). Therefore, the government’s epidemic prevention policies are constantly being adjusted and optimized according to changes in the epidemic situation.

The study takes the COVID-19 pandemic as the research context, constructing an explanatory framework that considers the impact of the stringency of epidemic prevention policies and risk perception on public vaccination willingness. Using multivariate linear regression models, it analyses the mechanism through which the stringency of epidemic prevention policies influences public vaccination willingness. The findings aim to provide a decision-making reference for active responses to the next wave of the urban infectious diseases, as well as informing vaccination strategies for the prevention and control of similar epidemics.

## 2 Literature review and research hypotheses

### 2.1 The health belief model

The Health Belief Model refers to the beliefs and behaviors that individuals adopt to protect their health status or promote their health to achieve self-fulfillment or self-actualization; it is mainly applied to predict how individuals will adopt a certain type of health behavior (15). The Health Belief Model contains six dimensions related to health behaviors: (1) perceived susceptibility, the individual’s perception of the probability that he or she will contract a certain disease; (2) perceived severity, the individual’s perception of the severity of the disease if he or she were to contract a certain disease; (3) perceived benefit, the individual’s judgment that performing or abandoning a certain behavior can alleviate the consequences of the disease that the individual has; (4) perceived barriers, the individual’s perception of certain difficulties, such as pain, faced in adopting the health behavior; (5) self-efficacy, the individual’s ability and confidence in his or her ability to carry out the health behavior; and (6) health motivation, the factors that can affect the individual’s ability to adopt the health behavior (16).

In the context of the COVID-19 pandemic, a group of researchers explored public vaccination willingness and risk perception using health belief modeling. Their study found that several factors, in addition to the technical or financial accessibility of vaccines, influence vaccine compliance. These factors are consistent with the Health Belief Model, which is widely recognized as the predominant theoretical framework utilized for forecasting vaccine adherence (17). The Health Belief Model emphasizes the influence of an individual’s beliefs and perceptions about health behaviors and measures, such as vaccination, on their health behaviors (18). These influences include concerns about vaccine effectiveness, safety, side effects, perceptions of the severity of the diseases that vaccines are designed to prevent, and susceptibility to vaccine infections (15). A substantial association was found between vaccination acceptance and the variables within the Health Belief Model. Significantly higher rates of vaccination acceptance were observed among respondents who held a perception of COVID-19 as a serious threat, acknowledged the benefits associated with the vaccine, and received cues to take action (19).

From the application of the fundamental elements of the Health Belief Model and empirical research, it is evident that demographic variables, psychosocial variables, and the perceived risks of vaccines are important influences on individuals’ adoption of health behaviors. Meanwhile, the Health Belief Model also includes dimensions of risk perception, namely perceived susceptibility and perceived severity, which can be incorporated into the questionnaire design to measure risk perception.

### 2.2 The stringency of epidemic prevention policies and vaccination willingness

Vaccination willingness refers to the acceptability of the vaccine, that is, whether an individual chooses to receive the vaccine to prevent a certain type of disease (20). Vaccination, as a key protective behavior, has attracted much attention. COVID-19 vaccination willingness has been found to have an impact on the prevalence of subsequent vaccination behaviors among the public and on the barrier components of herd immunity (21). At its essence, vaccination willingness is an epidemic prevention behavior. In 2019, the WHO identified vaccine hesitancy as one of the foremost top ten global health issues. Vaccine hesitancy, as defined by the Strategic Advisory Group of Experts (SAGE) on Immunization, refers to a delay in the acceptance or refusal of vaccination despite the availability of vaccination services (22). In existing research, the influence of various factors on public vaccination willingness has been analyzed to provide a scientific foundation and theoretical direction for the execution of targeted intervention.

A study was conducted to research public vaccination willingness among 2,006 adults in the United States regarding receiving the COVID-19 vaccine. The study found that interpersonal communication with medical workers, perceived susceptibility, perceived severity, and perceived effectiveness positively and significantly affected the willingness of respondents to be vaccinated (23). Moreover, a survey of 2,512 respondents in France found that age (older), gender (male), occupation (medical care), perceived susceptibility, and perceived severity had a positive and significant impact on public vaccination willingness (24). Higher level of education, good level of knowledge, previous history of COVID-19, male sex, and chronic disease were factors that positively affected the COVID-19 vaccine acceptance rate (25, 26). Males, individuals residing in Flanders, and those who tested positive for COVID-19 after receiving the first booster vaccine were more likely to receive the second booster vaccine (27). These findings highlight the potential influence of previous COVID-19 vaccination history and history of infection on individuals’ booster vaccine uptake. Through a systematic review, it was found that perceived risk, worries about the safety and effectiveness of the vaccine, and vaccination history were common factors affecting public COVID-19 vaccination willingness (25).

The government’s epidemic prevention policies have an important impact on people’s understanding of major public health emergencies and self-protection. In the face of emerging infectious diseases such as COVID-19, vaccination is the most important measure to avoid infection with COVID-19 and cut off the transmission path; it is the most effective protective behavior (13). When major public health emergencies occur, adjustment of the stringency of the government’s epidemic prevention policies enable the public to take more effective prevention measures based on their own knowledge. Studies have shown that the presence of both positive and negative government incentives is expected to enhance the probability of individuals getting vaccinated, with positive incentives potentially exerting a more pronounced influence on their inclination to vaccination (10). In the context of public health events, compliance with public protective measures is affected by many factors. Among them, the stringency of the government’s epidemic prevention policies is an important factor affecting the decision-making of individual protective behavior. The stricter the government’s epidemic prevention policies, the more inclined the public are to adopt protective measures, leading to a higher level of public vaccination willingness. Accordingly, the following hypothesis is proposed:

*Hypothesis 1:* The more stringent the government’s epidemic prevention policies, the stronger the public vaccination willingness.

### 2.3 Epidemic risk perception and vaccination willingness

Risk perception is a common term for describing people’s attitudes and intuitive assessments of risk, and it plays a key role in human behavior (28). After a major public health crisis, a fluctuation in public risk perception is observed due to the ambiguity surrounding the progression of the situation and the asymmetry of epidemic information. Various protective measures will be taken, such as obtaining relevant information on the epidemic and purchasing epidemic prevention items (29). In addition, risk perception significantly affects citizens’ response behavior and mental health. When individuals face events involving risk, the uncertainty associated with the risk and the resulting serious consequences contribute to heightened feelings of anxiety and panic. This can induce a state of depression, which increases public psychological pressure and makes it easier for individuals to engage in positive protective behavior to protect themselves (30). In the COVID-19 pandemic, the central and local government departments at all levels promptly issued pertinent policies and plans for epidemic prevention and control. The stricter the government’s epidemic prevention policies are, the higher the level of epidemic risk perceived by the public, prompting the public to take necessary measures to alleviate psychological pressure and avoid risk.

Previous research indicated that risk perception plays a critical role in shaping individuals’ acceptance of vaccines. According to several researchers, the level of social acceptance of COVID-19 vaccinations is influenced in a positive manner by individuals’ perception of risk (31). For example, we analyzed the factors that affect IGCV in American adults and reported that risk perception positively influences public intentions (32). A similar review of public acceptance of COVID-19 vaccines to prevent pandemic transmission found that perceptions of the risk of contracting acute illness persuaded individuals to get vaccinated (33). In addition, perceiving a high risk of infections may increase willingness and uptake rates for both testing and vaccination (34). Therefore, this study posits that when individuals are aware of their susceptibility to the epidemic and its severity, an elevated public perception of epidemic risk will lead to a faster public response. This, in turn, results in a reduced risk of infection through the implementation of more proactive, active, and stricter protective measures. Accordingly, the following hypotheses are proposed:

*Hypothesis 2:* The more stringent the government’s epidemic prevention policies are, the higher the public epidemic risk perception.

*Hypothesis 3:* The higher the public epidemic risk perception is, the stronger the public vaccination willingness.

### 2.4 The mediating role of risk perception in the relationship between the stringency of epidemic prevention policies and vaccination willingness

While more stringent government epidemic prevention policies may enhance public vaccination willingness, the mechanism of action remains unclear. Risk perception has an important influence on individual behavior in hazardous circumstances, and other social factors may indirectly affect individual protective behavior through the mediating role of risk perception (35). The Pressure–State–Response model contends that the relationship between external pressure, individual state, and individual response behavior forms an interactive decision-making process. The influence of external pressure on individual behavior is realized through an individual’s current perception and psychological state, which are both affected by pressure (36). The COVID-19 pandemic is a typical public health emergency, and the public’s willingness to protect themselves from the epidemic is a typical stress response to changes in their own risk perception caused by the pressure of virus transmission and prevention policies; this is a typical stress response process. The descriptive normative information received by the public during the period of risk may influence their perception and judgment of risk (37). The public’s attention to epidemic information mainly focuses on information about the government’s epidemic prevention measures and information related to the epidemic itself (38). Within the framework of the COVID-19 pandemic, the public’s perception of epidemic risk is shaped by governmental epidemic prevention measures and media reports. To mitigate the perceived risk, the public engage in measures such as vaccination and reduced socialization. Therefore, examining the effect of the stringency of government epidemic prevention policies on individual vaccination intentions from the perspective of risk perception helps in understanding the mechanism of action between these two factors. Accordingly, the following hypothesis is proposed:

*Hypothesis 4:* Public epidemic risk perception plays a mediating role in the effect of government epidemic prevention policy stringency on public vaccination willingness.

**Figure 1:**
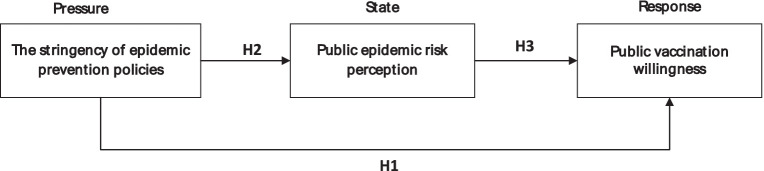
Research model.

In summary, based on the Health Belief Model and the Pressure–State–Response model, the research model and hypotheses of this study were constructed by taking the stringency of epidemic prevention policies as the independent variable, public vaccination willingness as the dependent variable, and public epidemic risk perception as the mediating variable (Figure 1).

## 3 Materials and methods

### 3.1 Survey procedures and participants

A cross-sectional design and convenience sampling method were used in this study. A cross-sectional survey was conducted from December 2022 to June 2023 to assess public vaccination willingness, the stringency of prevention policies, and risk perception regarding the COVID-19 pandemic in China. Due to the difficulties of conducting a face-to-face survey at the time of the COVID-19 pandemic, a combination of online and offline survey methods was used. To determine sample size, we used a single population proportion formula, taking into account a 95% level of confidence, a 5% sampling error or precision limit, and an assumed proportion of individuals willing to pay for the COVID-19 vaccine of 50%. The minimum sample size was determined to be 385, and the final sample size was 387.

The details of the questionnaire are shown in the Supplementary material. Two public health experts assessed and validated the survey instrument, providing several suggested modifications to improve the content and clarity. The author translated the questionnaire into Chinese, and two bilingual researchers ensured its clarity. The questionnaire was tested on a pilot sample to ensure its accuracy, after which two questions were modified to simplify their language.

### 3.2 Variable measurement and description

#### 3.2.1 Public vaccination willingness

The dependent variable is the public vaccination willingness. Referring to the well-established scales with similar variables in the previous studies (15–17, 21, 23, 25), public vaccination willingness was measured by asking the respondents the following question: “Would you be willing to receive the COVID-19 vaccine if it were available?” (Table 1). The responses were measured using a 5-point Likert scale, with a higher score on the scale indicating a stronger level of public vaccination willingness.

**Table 1:** Partial variable item description.

Variable	Dimension	Item	Description
Public vaccination willingness		PV	Would you be willing to receive the COVID-19 vaccine if it were available?
The stringency of epidemic prevention policies		SJD1	Requirement of not going out for nonessential purposes
		SJD2	24 h nucleic acid test for all staffs
		SJD3	Isolation of close contacts for 7/14 days
		SJD4	Showing health codes when entering and leaving public places
		SJD5	Catering businesses banning dine-in
		SJD6	Travel norms to wear masks
Public epidemic risk perception	Risk severity	FX1	I think once infected with COVID-19 will have a very serious impact on physical health
	Risk perceived susceptibility	FX2	I think many people are likely to be infected with COVID-19, including my family and friends
	Risk controllability	FX3	I think the epidemic and spread of the epidemic is difficult to control
	Risk fear	FX4	I think infected with COVID-19 will make me panic
	Vaccine safety and effectiveness	FX5	I doubt the effectiveness and safety of COVID-19 vaccine

#### 3.2.2 The stringency of epidemic prevention policies

The independent variable is the stringency of epidemic prevention policies. Referring to previous studies, many states implemented the WHO’s recommended precautions, including social and physical distancing, masking, hygiene practices, isolation of the ill, and quarantining cases of potential exposure (39). Based on Chinese official documents (40), this study placed Chinese government prevention measures into six categories, asking the respondents to make judgments on the degree of stringency of six government prevention measures during the COVID-19 pandemic (Table 1). This was measured on a 5-point Likert scale, with higher scores indicating more stringent epidemic prevention policies.

#### 3.2.3 Public epidemic risk perception

Many scholars have conducted research on the dimensional study of risk perception, such as the classic risk perception model, which demonstrates the familiarity and control dimensions of risk perception (41). However, the model is not applicable to the analysis of outbreaks because vaccination is essentially a disease-related health behavior that may require consideration of health factors, such as perceived susceptibility and perceived severity, in the dimensions of risk perception (16). On this basis, some studies have added other variables to measure risk perception more comprehensively, such as adding familiarity and control dimensions to the development of a scale for influenza outbreaks in public health emergencies to quantify risk perception more accurately (42).

Based on Slovic’s risk perception model and the Health Belief Model (19, 31, 32, 34, 41), this study comprehensively measured epidemic risk perception from five aspects: perceived susceptibility, severity, controllability, fear degree, vaccine safety, and effectiveness. The five dimensions of the corresponding questionnaire are shown in Table 1. The items were measured using a 5-point Likert scale, with higher scores indicating a higher level of public epidemic risk perception.

#### 3.2.4 Control variables

To accurately estimate the impact of the stringency of epidemic prevention policies on public vaccination willingness, individual characteristics such as gender, age, occupation, and education level were selected as control variables.

In addition, self-rated health refers to an individual’s subjective evaluation and expectation of his or her health status (43). Although self-rated health is a subjective indicator, it is often consistent with an individual’s objective physical health status (44–46). Differences in self-rated health may lead to varying degrees of willingness for individuals to adopt the same health behavior. Therefore, the heterogeneity between people with different levels of self-rated health will also have an impact on vaccination willingness. Different scholars have different settings for the measurement of self-assessed health, with the common international approach being to provide respondents with four or five alternative items to choose from based on their own health status (46–48). The authors of the current study also referred to Chinese national questionnaires such as the China Health and Retirement Longitudinal Study (CHARLS) and the Chinese General Social Survey (CGSS), setting the question “How do you feel about your current health status?” as the basis for examining respondents’ self-rated health, using a 5-point Likert scale with the following options: “very good,” “good,” “fair,” “bad,” and “very bad.”

### 3.3 Statistical analysis

In this study, data processing and analysis were conducted using SPSS 26.0 and Amos 23.0. First, exploratory factor analysis and confirmatory factor analysis were used to test the reliability and validity of the scale. In accordance with the principles of exploratory factor analysis, principal component analysis was applied as an extraction method to calculate the factor loading matrix, eigenvalue, contribution rate, and cumulative contribution rate of each indicator variable after Varimax rotation. Common method bias tests were performed using Harman’s single factor test (details are listed in Supplementary Table S1). Second, descriptive analysis was conducted to generate statistical summaries of the sample data, univariate analyses were conducted using Student’s *t* test and one-way ANOVA. Finally, a multiple linear regression model was used to study the effect of the stringency of epidemic prevention policies on public vaccination willingness. A hierarchical regression model was used to study the mediating effect of public risk perception, using the Bootstrap mediation test to examine the mediating effects.

## 4 Results

### 4.1 Reliability and validity test

Reliability refers to the consistency and stability of the survey results. Cronbach’s coefficient is the most common method for reliability analysis of the consistency of all index items in questionnaire surveys (49). A general coefficient value that reaches 0.7 shows that the internal consistency test of a scale is meaningful. As shown in Table 2, the Cronbach’s α coefficients of the variables were 0.89 (95% CI:0.85–0.88) and 0.75 (95% CI:0.74–0.788). The Cronbach’s α coefficients of the overall questionnaire was 0.87 (95% CI, 0.84–0.89). Since both values are greater than 0.70, this suggests that the questionnaire exhibits a high level of reliability and that it meets the research requirements.

**Table 2:** Measurement reliability and validity test results summary.

Variable	Item	Factor load coefficient	Cronbach’s α (95%CI)	CR	AVE	VAF
The stringency of epidemic prevention policies	SJD1	0.75	0.86 (0.85–0.88)	0.89	0.60	33.86
	SJD2	0.71				
	SJD3	0.82				
	SJD4	0.82				
	SJD5	0.77				
	SJD6	0.73				
Public epidemic risk perception	FX1	0.65	0.75 (0.74–0.78)	0.75	0.38	29.80
	FX2	0.54				
	FX3	0.66				
	FX4	0.59				
	FX5	0.62				

Before the validity test, the sample was tested for common method bias using the Harman’s single factor test. The results showed that the explanation rate of the first factor was 36.87% (details are listed in Supplementary Table S1), which was lower than the critical criterion of 40% (50), indicating that there was no serious common method bias in this study.

Validity is the degree of conformity between the results of a survey and the actual content of the investigation, that is, the degree to which the items to be measured can be accurately measured. Content validity and construct validity are both generally needed in research studies. Content validity indicates whether a scale effectively reflects the content being measured. Given that the dimensions and dimension measurement items in our study are grounded in a large number of literature analyses, it can be stated that the scale used is a feasible and universal health belief scale verified by practice. Therefore, the questionnaire of this study has good content validity.

Construct validity was mainly tested by KMO, Bartlett’s sphericity, and confirmatory factor analysis. First, KMO and Bartlett’s sphericity tests were conducted using SPSS to determine whether the collected data could be analyzed by factor analysis. The KMO test value of the questionnaire in this study was 0.89, exceeding the threshold of 0.70, and the Bartlett sphericity test results indicated a significance level < 0.05, indicating a correlation between the variables. These findings suggest that the data are suitable for identifying factor dimensions. In addition, the criterion of an eigenvalue equal to or greater than 1 was used for factor extraction, and all variables were extracted as expected. The results of the factor analysis showed that the cumulative variance contribution of the first two factors amounted to 58.68% (Table 2). Considering the nature of social science research, where the first two factors encompass most of the information from the original variables, the 11 evaluation indicators can be divided into two categories for study. Second, we tested the convergence validity using Amos 23.0. As shown in Table 2, the index factor load of each variable was found to be greater than 0.450, and the combined reliability (CR) was higher than 0.70. Although the average variance extraction (AVE) of the epidemic risk perception was less than 0.50, according to the relevant literature if the AVE is less than 0.50 but the comprehensive reliability is greater than 0.60, the convergence validity of the scale is still sufficient (50, 51).

### 4.2 Descriptive statistical analysis

Table 3 shows summary statistics on sociodemographic characteristics, including sex, age, education, occupation, and self-rated health. In terms of gender, there were about the same number of males and females, with 49.61% of the sample being male and 50.39% being female. Regarding age distribution, the largest group was 18–30 years old, accounting for 43.41% of the sample, followed by those who were 46–59 years old, accounting for 27.65% of the sample; the smallest number of respondents were over 60 years old, accounting for only 8.53% of the sample. In terms of education, more than half of the respondents had a bachelor’s degree from a university, accounting for 63.05% of the sample, approximately 19.64% had a master’s degree or above, and only 17.31% had completed high school, reflecting the high level of education of the respondents of this survey. In terms of occupation, the largest proportion of respondents were in the student group, accounting for 29.20% of the sample, followed by enterprise personnel and self-employed individuals, accounting for 27.13 and 19.90%, respectively, while civil servants and institutions accounted for only 12.14%, probably due to the limitation of the distribution scope of the questionnaire. In terms of self-rated health, most of the respondents were satisfied with their own health status, 64.90% thought that they had good health status, and only 9.56% of the respondents perceived their health status to be poor, while 25.6% of the sample perceived their health status to be average, accounting for 25.58% of the sample.

**Table 3:** Descriptive statistics of sample characteristics (*n* = 387).

Demographic Factors	Classification	*N*	Proportion
Sex	Male	192	49.61%
	Female	195	50.39%
Age	18–30	168	43.41%
	31–45	79	20.41%
	46–59	107	27.65%
	>60	33	8.53%
Occupation	Student	113	29.20%
	Government/institution staff	47	12.14%
	Workers of enterprise	105	27.13%
	business	77	19.90%
	Other	45	11.63%
Education level	Senior high school and below	67	17.31%
	University	244	63.05%
	Graduate and above	76	19.64%
Self-rated health	Very good	70	18.09%
	Good	181	46.77%
	Average	99	25.58%
	Bad	29	7.49%
	Very bad	8	2.07%

In addition, the mean score of the stringency of epidemic prevention policies was found to be high, with an average score of 3.83 ± 0.46, indicating that the respondents generally believed that the government’s epidemic prevention policies were highly stringent. Risk perception had an average score of 3.56 ± 0.26, indicating that the respondents had a certain level of risk perception, while public vaccination willingness had an average score of 3.68 ± 0.57, supporting that the majority of the respondents were willing to be vaccinated.

### 4.3 Differential analysis of epidemic prevention policy stringency, risk perception, and vaccination willingness among different populations

The population was divided into different groups based on the results of demographic variable analysis to analyze the differences in epidemic prevention policy stringency, risk perception, and vaccination willingness, including differences in variables such as gender, age, and occupation. If the population was divided into two groups, an independent samples *t*-test was used; if the population was greater than or equal to three groups, a one-way ANOVA was used, and when the ANOVA chi-square test failed, the Welch method was used for correction. Specifically, Student’s *t* test and ANOVA were used as the variables of interest conformed to a normal distribution and passed the chi-square test (details are listed in Supplementary Tables S2–S8).

First, independent samples *t*-tests were performed on different gender groups. There was a significant gender difference in epidemic risk perception (*p* = 0.041), women specifically exhibited a higher perception of epidemic risk than men. Furthermore, no empirical evidence supported a significant gender difference in the stringency of epidemic prevention policies and vaccination willingness between different genders (Table 4). Second, ANOVA analyses were performed on different age groups. The results supported that there was a significant age difference in epidemic risk perception and vaccination willingness (*p* = 0.012, *p* = 0.016), with people over 60 years old showing the strongest effect (Table 5). Third, ANOVA analyses were used on groups with different education levels. The results supported that there was a significant educational difference in the stringency of epidemic prevention policy and vaccination willingness (*p* = 0.004, *p* = 0.040), among which groups with high levels of education exhibited the lowest levels of vaccination willingness (Table 6).

**Table 4:** The Student’s *t*-test of Gender (*n* = 387).

Variable	Male(192)		Female(195)		*t*	*p*
	MD	SE	MD	SE		
The stringency of epidemic prevention policies	3.77	0.74	3.89	0.74	1.54	0.126
Public epidemic risk perception	3.49	0.65	3.63	0.69	2.05	0.041*
Public vaccination willingness	3.65	0.80	3.71	0.84	0.74	0.458

*p < 0.05, **p < 0.01, ***p < 0.001.

**Table 5:** One-way ANOVA of age (*n* = 387).

Variable		MD	SE	F	*p*
The stringency of epidemic prevention policies	18–30	3.56	0.74		
	31–45	3.71	0.59		
	46–59	3.84	0.84		
	>60	3.96	0.71		
	Between-group			1.113	0.344
Public epidemic risk perception	18–30	3.38	0.65		
	31–45	3.62	0.57		
	46–59	3.68	0.69		
	>60	3.93	0.70		
	Between-group			9.082	0.012*
Public vaccination willingness	18–30	3.57	0.83		
	31–45	3.71	0.83		
	46–59	3.74	0.79		
	>60	3.89	0.82		
	Between-group			9.324	0.016*

*p < 0.05, **p < 0.01, ***p < 0.001.

**Table 6:** One-way ANOVA of education level (*n* = 387).

Variable		MD	SE	F	*p*
The stringency of epidemic prevention policies	Senior high school and below	3.10	0.69		
	University	3.76	0.76		
	Graduate and above between-group	3.83	0.68		
				5.708	0.004**
Public epidemic risk perception	Senior high school and below	3.85	0.81		
	University	3.51	0.64		
	Graduate and above	3.47	0.57		
	Between-group			2.250	0.184
Public vaccination willingness	Senior high school and below	3.90	0.72		
	University	3.61	0.82		
	Graduate and above	3.50	0.86		
	Between-group			8.101	0.040*

*p < 0.05, **p < 0.01, ***p < 0.001.

### 4.4 The direct effect of the stringency of epidemic prevention policies on public vaccination willingness

The COVID-19 pandemic has significantly affected the physical and mental well-being, as well as the behavioral patterns, of the general population. In the face of the challenges posed by the COVID-19 pandemic, the government has taken active measures to ensure the safety and health of the people across the country. Based on China’s special national conditions and the public trust in the government, the government’s epidemic prevention policies have had an important impact on public vaccination willingness. Since the dependent variable is a continuous variable, OLS linear regression was used in this study. VIF < 10 in the model covariance diagnostics, so there is no multicollinearity. Table 7 shows the net effect of the stringency of epidemic prevention policies on public vaccination willingness. To better evaluate the impact of the stringency of epidemic prevention policies on public vaccination willingness, the following regression strategies were adopted: first, Model 1 was obtained by considering the impact of the stringency of epidemic prevention policies on public vaccination willingness; second, Model 2 was obtained by adding individual characteristic variables that may affect the vaccination willingness based on Model 1.

**Table 7:** OLS Multiple linear regression of the stringency of epidemic prevention policies on public vaccination willingness and mediating effect of public epidemic risk perception.

Variable	Public vaccination willingness	Public vaccination willingness	Public epidemic risk perception	Public vaccination willingness	Public vaccination willingness
	Model 1	Model 2	Model 3	Model 4	Model 5
The stringency of epidemic prevention policies	0.534***	0.642**	0.389**		0.455***
	[0.496–0.683]	[0.504–0.694]	[0.309–0.469]		[0.399–0.606]
	(0.000)	(0.002)	(0.003)		(0.000)
Public epidemic risk perception				0.409***	0.203***
				[0.381–0.615]	[0.131–0.365]
				(0.000)	(0.000)
Age		0.188*	0.164**	−0.131*	0.121**
		[0.151–0.270]	[0.039–0.175]	[−0.193–0.016]	[0.106–0.317]
		(0.027)	(0.002)	(0.021)	(0.017)
Education		0.152**	−0.031	0.142*	0.263**
		[0.123–0.165]	[−0.138–0.070]	[0.101–0.277]	[0.112–0.436]
		(0.003)	(0.519)	(0.032)	(0.002)
Occupation		0.103	0.122	−0.004	0.078
		[−0.001–0.124]	[0.007–0.212]	[−0.070–0.065]	[0.015–0.108]
		(0.053)	(0.086)	(0.948)	(0.135)
Self-rated health		0.181*	0.102*	0.057	0.032*
		[0.168–0.296]	[0.034–0.196]	[0.034–0.134]	[−0.104–0.047]
		(0.024)	(0.034)	(0.239)	(0.015)
_cons	1.416***	1.472**	1.581**	2.452**	1.080***
	(1.051–1.781)	[0.888–2.057]	[1.089–2.073]	[1.862–3.043]	[0.479–1.682]
	(0.000)	(0.004)	(0.002)	(0.003)	(0.000)
F	153.65***	31.782***	26.210***	14.876***	30.502***
Adjusted R-squared	0.283	0.294	0.246	0.252	0.314
*N*	387	387	387	387	387

*p < 0.05, **p < 0.01, ***p < 0.001.

The above two models showed that the coefficient of the stringency of epidemic prevention policies was positive, passing the significance level of 1%. After controlling for the influence of other factors, the coefficient of Model 2 was found to be higher than that of Model 1 (+ 0.108), and the overall explanatory power of the model was enhanced (+ 0.011), indicating that the estimation results of Model 2 were more valuable. Therefore, Model 2 showed that for every one-unit increase in the stringency of epidemic prevention policies, public vaccination willingness increased by approximately 64.2% (R2 = 0.294, b = 0.642, 95%CI: [0.504–0.694], *p* < 0.001). Thus, the stringency of epidemic prevention policies exerts a substantial beneficial influence on public vaccination willingness. Based on the above results, accepting Hypothesis 1; that is, the more stringent the epidemic prevention policies are, the stronger the public vaccination willingness.

In addition, a comprehensive observation of the estimated results of the control variables indicated a notable positive link between the level of education and public vaccination willingness (b = 0.152, 95%CI: [0.123–0.165], *p* = 0.003). The higher the level of education, the more information and knowledge residents can obtain about the epidemic. Therefore, in the face of large-scale outbreaks of infectious diseases, effective self-protection measures are taken (52), such as vaccination with new coronavirus vaccines. There was also a significant correlation between age and public vaccination willingness, with people of different ages displaying differing levels of vaccination willingness (b = 0.188, 95%CI: [0.151–0.270], *p* = 0.027). Simultaneously, a notable positive association was found between individuals’ state of health and public vaccination willingness (b = 0.181, 95%CI: [0.168–0.296], *p* = 0.024). The public’s evaluation and perception of their own health status affects their choice of protective behavior in the face of the government’s epidemic prevention policies. No correlation was found between occupation and public vaccination willingness.

### 4.5 The mechanism of the impact of the stringency of epidemic prevention policies on public vaccination willingness

The previous section supported that the stringency of government epidemic prevention policies has indeed significantly increased public vaccination willingness. This paper argues that as the most direct factor affecting public vaccination willingness, public risk perception plays an essential mediating role between the stringency of epidemic prevention policies and public vaccination willingness. The stringency of epidemic prevention policies affects vaccination willingness through risk perception. A stepwise test method was used to examine the mediating effect of risk perception between the stringency of epidemic prevention policies and vaccination willingness (53). Specifically, on the basis of the previously observed direct effects, the following steps were carried out: first, testing whether the stringency of epidemic prevention policies has a significant impact on vaccination willingness; second, testing whether the impact of risk perception on vaccination willingness is significant; third, if the tests of the first two steps were passed, to continue to test whether the stringency of epidemic prevention policies and risk perception have a significant impact on vaccination willingness. If the impact of the stringency of epidemic prevention policies on vaccination willingness weakened after adding the wind perception variable, then the mediating effect of risk perception would be established.

Table 7 reports the impact mechanism of the stringency of epidemic prevention policies on vaccination willingness. The results from Model 3 showed that the stringency of epidemic prevention policies has a significant positive impact on public risk perception. Specifically, the respondents’ risk perception level increased by approximately 38.9% during the strict period of government epidemic prevention policies (b = 0.389, 95%CI: [0.309–0.469], *p* < 0.001). Thus, Hypothesis 2 is verified; the more stringent the government’s epidemic prevention policies are, the higher the public’s perception of risk. The results from Model 4 showed that for each additional unit of risk perception, public vaccination willingness increased by approximately 45.5%, and the overall explanatory power of the model reached 31% (R2 = 0.316, b = 0.455, 95%CI: [0.399–0.606], *p* < 0.001). Thus, Hypothesis 3 is verified; the higher the risk perceived by the public, the stronger the willingness to vaccinate. Incorporating the stringency of epidemic prevention policies and risk perception into the regression model, it was found that compared with Model 2, the overall explanation of Model 5 was significantly enhanced (+0.04). This indicates that risk perception is a key factor affecting public vaccination willingness.

At the same time, compared with Model 2, the coefficient of the stringency of epidemic prevention policies in Model 5 decreased significantly (−0.187). According to the criteria of Baron et al. (53), risk perception plays a partial intermediary role between the stringency of epidemic prevention and control policies and public vaccination willingness. This indicates that the stringency of the government’s epidemic prevention policies affects vaccination willingness through public risk perception, preliminarily verifying the existence of risk perception as an intermediary variable. Thus, Hypothesis 4 is preliminarily verified.

**Figure 2:**
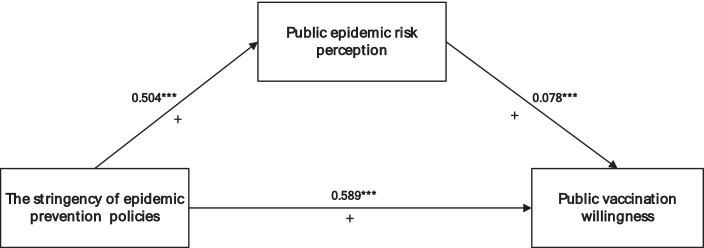
The theoretical model of public epidemic risk perception as a mediator.

This study completed the test of the mediating effect with the help of Model 4 in the SPSS macro program PROCESS compiled by Hayes; the results are shown in Table 8. The results support that from the intermediary path of epidemic prevention policy stringency → public epidemic risk perception → public vaccination willingness, bootstrap 95% confidence intervals do not contain 0, and an intermediary path exists, indicating that risk perception has a substantial moderating impact on the effect of epidemic prevention policy stringency on vaccination willingness. This further confirms H4. The influence mechanism of the stringency of epidemic prevention policies on public vaccination willingness is shown in Figure 2.

**Table 8:** Bootstrap estimates of the mediating effect of public epidemic risk perception.

	Effect	SE	LLCI	ULCI
Mediating effect	0.078***	0.024	0.033	0.132
Directing effect	0.504***	0.051	0.402	0.608
Total effect	0.589***	0.048	0.496	0.682

*p < 0.05, **p < 0.01, ***p < 0.001.

## 5 Discussion and conclusion

Considering the current state of epidemic prevention and control, vaccination is currently the most cost-effective prevention measure globally recognized in the fight against the COVID-19 pandemic (54), and the vaccine uptake of individuals is significantly influenced by the preventive measures implemented by governmental authorities. Encouraging the public to vaccinate is the key policy and means to improve group immunity and reduce the risk of viral infection (55). Thus, it is of paramount importance to thoroughly investigate the influencing factors of public vaccination willingness.

Existing research findings have focused on the factors affecting public vaccination willingness, such as good knowledge about vaccines, higher educational level, previous seasonal flu vaccination, female sex, and history of COVID-19 infection (56). It is worth noting that having a history of COVID-19 infection increases the acceptance of COVID-19 vaccination (25–27), but another study revealed that those who have never been infected with COVID-19 were more willing to receive the vaccine (57). A research survey in Bangladesh showed that the participants who had been vaccinated for other diseases after the age of 18 years old were 0.48 times less likely to have hesitancy towards the COVID-19 vaccine compared to those who had not been vaccinated after the age of 18 years (58). However, the impact of the stringency of government epidemic prevention policies on public willingness vaccination remains an open topic. How to open the “epidemic prevention and control policy stringency–vaccination willingness” black box is the problem that this article attempts to solve.

In our study, empirical evidence supports the positive association between the stringency of epidemic prevention policies and public vaccination willingness. As China entered the high-risk period of the COVID-19 pandemic, epidemic control measures become gradually stricter, and willingness to have the COVID-19 vaccination appeared to become relatively high. The findings are aligned with a few previous studies (10, 13, 14) reporting that with the evolution of the pandemic and changes in policy stringency, public vaccination willingness may change over time. Under the impact of the COVID-19 pandemic, the public is prone to excessive stress responses. From a psychological perspective, the promulgation of the government’s epidemic prevention measures is likely to cause public panic and anxiety, resulting in them taking actions to avoid risks, such as vaccination (14). Under the combined effect of the above two factors, the stricter the government’s epidemic prevention policies are, the more obvious the public’s stress response in both psychological and behavioral aspects. This is more likely to reduce the impact of the epidemic through vaccination.

Risk perception serves as a mediating factor between the stringency of epidemic prevention policies and public vaccination willingness. More specifically, the stricter the government’s epidemic prevention policies, the higher risk perceived by the public, and the stronger the willingness to vaccinate. These findings are coherent with previous studies (35, 36, 38) showing that in areas where the epidemic is more serious, heightened stringency of the government’s epidemic prevention measures lead to more negative information being released due to a ripple effect. This, in turn, results in a higher perceived risk by the public. Furthermore, the higher the perceived risk by the public, the more likely they are to reduce negative emotions and injuries by adopting certain measures, manifested as epidemic prevention behaviors such as vaccination. This is consistent with the results of a previous survey from China showing that there was a high willingness to be vaccinated against COVID-19 at the beginning of the pandemic, which declined as the pandemic became normalized due to the reduced perception of COVID-19 risk among the public (59). On the basis of the two preceding findings, this study supports that public risk perception mediates the relationship between the stringency of epidemic prevention policies and public vaccination willingness. The stringency of epidemic prevention policies affects public vaccination willingness through risk perception, thus opening the “epidemic prevention policies–vaccination willingness” black box.

In addition, higher awareness of and perceived susceptibility to COVID-19 were found to be positively associated with public vaccination willingness, which aligns with previous results reporting that perceiving a high risk of infection may increase vaccination willingness (25, 34). Moreover, a global survey reported that concerns regarding the efficacy and safety of the COVID-19 vaccine may be another reason for individuals’ lack of willingness to receive a vaccination (55).

This study tested the effects of control variables on vaccination willingness, which aligned with previous studies conducted in other countries (24, 25, 27, 34). However, international studies indicate that the willingness to receive the COVID-19 vaccine varies considerably according to socio-demographic characteristics, including gender and education, with age having a significant influence. Notably, the lowest levels of vaccination willingness are found among young adults (26, 60). In the Netherlands, the percentage of adults between 18 and 34 years who are willing to receive the COVID-19 vaccine constantly lies about 10 percentage points below the average percentage of the whole population (61). These findings indicate that young adults are the mainstay of vaccine hesitancy; therefore, a survey targeting young respondents aged 18–30 years, identifying factors that determine vaccination willingness among young adults would be highly valuable.

Furthermore, it is also noteworthy that vaccine adherence was higher in workplace vaccination campaigns, which could have been an interesting strategy to adopt and is consistent with earlier research (6). This probably occurred due to public health workers during the COVID-19 pandemic being at increased risk of violence and harassment due to their public health work; they experienced adverse mental health conditions. Ongoing training, workplace support, and enhanced communication after a workplace violence incident may be helpful (62).

### 5.1 Measures to enhance public vaccination willingness

This paper argues that enhancing public vaccination willingness can start by adjusting the stringency of epidemic prevention policies and improving public awareness of the risks, so as to establish a society-wide immunization barrier.

It is recommended to adjust the stringency of epidemic prevention policies in a timely manner. The stricter the epidemic prevention policies are, the stronger the public vaccination willingness. Accordingly, the stringency of prevention policies should be modified in response to changes in the epidemic prevention and control environment. More stringent prevention measures can be taken in areas with low vaccination rates, increasing the cost of epidemic prevention for the population, and thus stimulating the population to vaccinate and achieving the goal of establishing universal immunization (63). In addition, differentiated guidance to encourage vaccination willingness among different people should be implemented. The government should respect the heterogeneity and subjective preferences of different people and formulate differentiated vaccine promotion strategies for groups with weak vaccination willingness on the basis of the aforementioned publicity methods and policy support (10, 14, 27).

Public awareness of risk should also be increased. Risk perception serves as a mediating factor between the stringency of epidemic prevention policies and public vaccination willingness. The government should issue authoritative statements to publicize the complexity and severity of the international epidemic prevention situation, guiding individual risk perception. Based on the statements issued by authoritative institutions and experts, the risk information communication should be strengthened and the necessity of vaccination should be actively promoted, thus changing the public’s cognitive bias and resistance to COVID-19 vaccination (26, 29). By emphasizing the concept of a community with a shared future for humankind and the severity of the global epidemic prevention situation, the public can be guided to maintain a high level of epidemic risk perception (13).

### 5.2 Limitations

There are some limitations of the current study that need to be considered when interpreting the results. First, we used convenience sampling to collect data, and the number of participants was relatively low, which may result in sampling bias and lower the generalizability of the present findings. Second, while anonymity was used to minimize social desirability bias, social desirability bias may still exist. Since COVID-19 vaccination was the official strategy in China, it may be considered socially desirable to be vaccinated, which may result in an overestimation of the level of public vaccination willingness. It may be possible to eliminate the influence of social desirability response bias through using alternative methodologies such as randomized response methods, forced-choice items, proxy subjects, computer administration, and the BIDR scale (64, 65). Third, the cross-sectional design precludes causal or temporal inferences; the findings should be validated by longitudinal studies.

## Data availability statement

The raw data supporting the conclusions of this article will be made available by the authors, without undue reservation.

## Ethics statement

The studies involving humans were approved by Wuhan University of Technology. The studies were conducted in accordance with the local legislation and institutional requirements. Written informed consent for participation in this study was provided by the participants themselves. Written informed consent was obtained from the individuals for the publication of any potentially identifiable images or data included in this article.

## Author contributions

JZ: Conceptualization, Data curation, Formal analysis, Investigation, Methodology, Writing – original draft, Writing – review & editing, Software, Visualization. YZ: Funding acquisition, Methodology, Resources, Writing – review & editing, Conceptualization, Validation, Supervision. MZ: Writing – original draft, Data curation, Formal analysis, Investigation.
