# Supplementary material for: Impact of epidemic prevention policies on public vaccination willingness: empirical research in China
Source: Front Public Health. 2024 Jul 3;12:1329228. doi: 10.3389/fpubh.2024.1329228 (PMC11252039; doi:10.3389/fpubh.2024.1329228)
Supplement: Supplementary file 1 [file Data_Sheet_1.docx]

Supplementary Material

# Supplementary Figures

## Supplementary Figure 1. Research model.

**1.2** **Supplementary Figure 2.** The theoretical model of public epidemic risk perception as a mediator.

# Supplementary Tables

**Table 1** The Harman's single factor test

| **Component** | **Initial Eigenvalues** | | | **Extraction Sums of Squared Loadings** | | |
| --- | --- | --- | --- | --- | --- | --- |
|  | Total | % of Variance | Cumulative % | Total | % of Variance | Cumulative % |
| 1 | 3.825 | 36.865 | 36.865 | 3.825 | 36.865 | 36.865 |
| 2 | 1.849 | 21.811 | 58.676 | 1.849 | 21.811 | 58.676 |
| 3 | .674 | 7.131 | 65.807 |  |  |  |
| 4 | .652 | 6.923 | 72.730 |  |  |  |
| 5 | .581 | 6.283 | 79.013 |  |  |  |
| 6 | .556 | 5.053 | 85.066 |  |  |  |
| 7 | .492 | 4.468 | 89.534 |  |  |  |
| 8 | .420 | 3.814 | 93.348 |  |  |  |
| 9 | .369 | 2.351 | 95.699 |  |  |  |
| 10 | .319 | 2.879 | 98.578 |  |  |  |
| 11 | .264 | 1.422 | 100 |  |  |  |

**Table 2** Evaluation of the variables of interest Normality on gender

| **Variables** |  | **Kolmogorov-Smirnov(n>50)** | | | **Shapiro-Wilk(n>50)** | | |
| --- | --- | --- | --- | --- | --- | --- | --- |
|  |  | Statistic | df | p | Statistic | df | p |
| SDJ | Male | .102 | 192 | .214 | .951 | 192 | .479 |
|  | Female | .113 | 195 | .201 | .942 | 195 | .431 |
| FX | Male | .113 | 192 | .206 | .973 | 192 | .590 |
|  | Female | .102 | 195 | .189 | .973 | 195 | .495 |
| PV | Male | .239 | 192 | .356 | .861 | 192 | .324 |
|  | Female | .257 | 195 | .389 | .870 | 195 | .358 |

**Table 3** Evaluation of the variables of interest Normality on age

| **Variables** |  | **Kolmogorov-Smirnov(n>50)** | | | **Shapiro-Wilk(n>50)** | | |
| --- | --- | --- | --- | --- | --- | --- | --- |
|  |  | Statistic | df | p | Statistic | df | p |
| SDJ | 18-30 | .227 | 168 | .201 | .864 | 168 | .302 |
|  | 31-45 | .257 | 79 | .211 | .866 | 79 | .305 |
|  | 46-59 | .249 | 107 | .218 | .859 | 107 | .301 |
|  | >60 | .329 | 33 | .253 | .824 | 33 | .294 |
| FX | 18-30 | .127 | 168 | .102 | .943 | 168 | .389 |
|  | 31-45 | .121 | 79 | .106 | .962 | 79 | .418 |
|  | 46-59 | .129 | 107 | .100 | .921 | 107 | .423 |
|  | >60 | .247 | 33 | .102 | .719 | 33 | .305 |
| PV | 18-30 | .121 | 168 | .145 | .964 | 168 | .424 |
|  | 31-45 | .146 | 79 | .143 | .959 | 79 | .425 |
|  | 46-59 | .117 | 107 | .144 | .946 | 107 | .424 |
|  | >60 | .265 | 33 | .143 | .755 | 33 | .123 |

**Table 4** Evaluation of the variables of interest Normality on education

| **Variables** |  | **Kolmogorov-Smirnov(n>50)** | | | **Shapiro-Wilk(n<50)** | | |
| --- | --- | --- | --- | --- | --- | --- | --- |
|  |  | Statistic | df | p | Statistic | df | p |
| SDJ | Senior high school and below | .349 | 67 | .213 | .769 | 67 | .345 |
|  | University | .240 | 244 | .223 | .867 | 244 | .369 |
|  | Graduate and above | .251 | 76 | .218 | .854 | 76 | .374 |
| FX | Senior high school and below | .209 | 67 | .156 | .874 | 67 | .456 |
|  | University | .098 | 244 | .089 | .947 | 244 | .458 |
|  | Graduate and above | .132 | 76 | .102 | .951 | 76 | .461 |
| PV | Senior high school and below | .159 | 67 | .213 | .936 | 67 | .586 |
|  | University | .107 | 244 | .204 | .975 | 244 | .532 |
|  | Graduate and above | .155 | 76 | .215 | .963 | 76 | .548 |

**Table 5** Evaluation of the equality of variances between times (Levene's test)

| **Test of Homogeneity of Variances on Age** | | | | | |
| --- | --- | --- | --- | --- | --- |
| **Variables** |  | Levene Statistic | df1 | df2 | p |
| SDJ | Based on Mean | 3.890 | 3 | 383 | .604 |
| FX | Based on Mean | .555 | 3 | 383 | .645 |
| PV | Based on Mean | .403 | 3 | 383 | .751 |

**Table 6** Evaluation of the equality of variances between times (Levene's test)

| **Test of Homogeneity of Variances on Education** | | | | | |
| --- | --- | --- | --- | --- | --- |
| **Variables** |  | Levene Statistic | df1 | df2 | p |
| SDJ | Based on Mean | 2.294 | 2 | 384 | .102 |
| FX | Based on Mean | 5.068 | 2 | 384 | .106 |
| PV | Based on Mean | 9.046 | 2 | 384 | .179 |

**Table 7** Evaluation of the equality of variances between times (Levene's test)

| **Test of Homogeneity of Variances on Health** | | | | | |
| --- | --- | --- | --- | --- | --- |
| **Variables** |  | Levene Statistic | df1 | df2 | p |
| SDJ | Based on Mean | 3.605 | 4 | 382 | .107 |
| FX | Based on Mean | .578 | 4 | 382 | .679 |
| PV | Based on Mean | .575 | 4 | 382 | .681 |

**Table 8** Multicollinearity and Durbin-Watson test

| **variables** | **Collinearity Statistics** | | **Durbin-Watson** |
| --- | --- | --- | --- |
|  | Tolerance | VIF |  |
| The stringency of epidemic prevention policies | 0.781 | 1.280 |  |
| Public epidemic risk perception | 0.744 | 1.344 |  |
| age | 0.688 | 1.454 |  |
| Occupation | 0.646 | 1.547 |  |
| education | 0.849 | 1.178 |  |
| Self-rated health | 0.953 | 1.049 |  |
| Total |  |  | 1.692 |
