# Supplementary material for: Impact of epidemic prevention policies on public vaccination willingness: empirical research in China
Source: Front Public Health. 2024 Jul 3;12:1329228. doi: 10.3389/fpubh.2024.1329228 (PMC11252039; doi:10.3389/fpubh.2024.1329228)
Supplement: Supplementary file 2 [file Data_Sheet_2.docx]

**Questionnaire of Epidemic prevention policies impact on public vaccination willingness**

**PART ONE: BACKGROUND INFORMATION**

**1. Sex:**

A. Male

B. Female

**2. Age:**

A. 18-30 years old

B. 30-45 years old

C. 45-59 years old

D. ≥60 years old

**3. Occupation:**

A. Students

B. Civil servants and public institution personnel

C. Enterprise personnel

D. Self-employed

E. Others

**4. Educational Attainment:**

A. High school or below

B. University degree

C. Master's degree or above

**5. District of Residence**: ( )

**6. Generally speaking, how do you feel about your current health status?**

A. Very good

B. Good

C. Fair

D. Bad

E. Very bad

**7. Is the government's epidemic prevention policy the main channel for you to learn about the COVID-19 outbreak?**

A. Yes

B. No

C. I do not know

**PART TWO: STRNGENCY OF EPIDEMIC PREVENTION POLICIES**

**8. In this section, we would like to learn a bit about your views on the stringency of the following epidemic prevention measures during the COVID-19 pandemic.**

| The following questions describe the stringency of government epidemic prevention measures: |
| --- |
| - 1. Requirement of not going out for nonessential purposes   A. Very loose B. Loose C. Average D. Strict E. Very strict |
| - 1. 24 h nucleic acid test for all staffs   A. Very loose B. Loose C. Average D. Strict E. Very strict |
| - 1. Isolation of close contacts for 7/14 days   A. Very loose B. Loose C. Average D. Strict E. Very strict |
| - 1. Showing health codes when entering and leaving public places   A. Very loose B. Loose C. Average D. Strict E. Very strict |
| - 1. Catering businesses banning dine-in   A. Very loose B. Loose C. Average D. Strict E. Very strict |
| - 1. Travel norms to wear masks   A. Very loose B. Loose C. Average D. Strict E. Very strict |

**PART THREE: PUBLIC EPIDEMIC RISK PERCEPTION**

**9.** **In this section, we would like to learn a bit about your overall risk perception of the COVID-19 epidemic.**

| The following questions describe the risk perception of the COVID-19 epidemic: |
| --- |
| - 1. I think once infected with COVID-19 will have a very serious impact on physical health   A. Totally disagree B. Disagree C. Neutrality D. Agree E. Totally agree |
| 9.2 I think many people are likely to be infected with COVID-19, including my family and friends  A. Totally disagree B. Disagree C. Neutrality D. Agree E. Totally agree |
| 9.3 I think the epidemic and spread of the epidemic is difficult to control  A. Totally disagree B. Disagree C. Neutrality D. Agree E. Totally agree |
| - 1. I think the epidemic and spread of the epidemic is difficult to control   A. Totally disagree B. Disagree C. Neutrality D. Agree E. Totally agree |
| - 1. I think infected with COVID-19 will make me panic   A. Totally disagree B. Disagree C. Neutrality D. Agree E. Totally agree |
| - 1. I doubt the effectiveness and safety of COVID-19 vaccine   A. Totally disagree B. Disagree C. Neutrality D. Agree E. Totally agree |

**PART FOUR: PUBLIC VACCINATION WILLINGNESS**

**10. In the context of the COVID-19 epidemic, would you be willing to receive the COVID-19 vaccine if it were available?**

A. Impossible

B. Unlikely

C. Not sure

D. Likely

E. Very likely
